# *Helicoverpa armigera* miR-2055 regulates lipid metabolism via fatty acid synthase expression

**DOI:** 10.1098/rsob.210307

**Published:** 2022-03-02

**Authors:** Yang Cheng, Tengfei Lu, Junliang Guo, Zhe Lin, Qiao Jin, Xiaoming Zhang, Zhen Zou

**Affiliations:** ^1^ State Key Laboratory of Integrated Management of Pest Insects and Rodents, Institute of Zoology, Chinese Academy of Sciences, Beijing, People's Republic of China; ^2^ College of Bioscience and Biotechnology, Yangzhou University, Yangzhou, People's Republic of China; ^3^ CAS Center for Excellence in Biotic Interactions, University of Chinese Academy of Sciences, Beijing, People's Republic of China; ^4^ Institute of Physical Science and Information Technology, Anhui University, Hefei, People's Republic of China

**Keywords:** *Helicoverpa armigera*, lipid metabolism, microRNA, juvenile hormone, development

## Abstract

Insect hormones and microRNAs regulate lipid metabolism, but the mechanisms are not fully elucidated. Here, we found that cotton bollworm larvae feeding on *Arabidopsis thaliana* (AT) leaves had a lower triacylglycerol (TAG) level and more delayed development than individuals feeding on artificial diet (AD). Association analysis of small RNA and mRNA revealed that the level of miR-2055, a microRNA related to lipid metabolism, was significantly higher in larvae feeding on AT. Dual-luciferase reporter assays demonstrated miR-2055 binding to 3′ UTR of fatty acid synthase (FAS) mRNA to suppress its expression. Elevating the level of miR-2055 in larvae by agomir injection decreased FAS mRNA and protein levels, which resulted in reduction of free fatty acid (FFA) and TAG in fat body. Interestingly, *in vitro* assays illustrated that juvenile hormone (JH) increased miR-2055 accumulation in a dosage-dependent manner, whereas knockdown of Methoprene tolerant (Met) or Kruppel homologue 1 (Kr-h1) decreased the miR-2055 level. This implied that JH induces the expression of miR-2055 via a Met-Kr-h1 signal. These findings demonstrate that JH and miRNA cooperate to modulate lipid synthesis, which provides new insights into the regulatory mechanisms of metabolism in insects.

## Graphical abstract

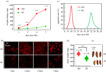

## 1. Introduction

Lipids are essential energy sources in animals and play important roles in individual growth and development. Therefore, lipid metabolism needs to be regulated precisely. Triacylglycerol (TAG) is the main component of lipid, and its principal biological functions have been highly conserved during evolution in metazoans [1].

In *Drosophila melanogaster*, TAG is hydrolysed into free fatty acids (FFAs) and glycerol under the control of two different lipases, Brummer (Bmm) and hormone-sensitive lipase (Hsl) [2,3], and FFAs can serve as substances in β-oxidation and other biochemical reactions. The regulation of TAG catabolism is strictly supervised by the insulin and adipokinetic hormone (AKH) pathways. AKH can promote the expression of protein kinase A (PKA) and phosphorylate perilipin-related protein 1(Plin1), which inhibits the activity of Bmm [4,5]. Insulin acts as an activator of protein kinase B (PKB), which can repress the activity of Bmm by phosphorylating transcription factor Orkhead box subgroup O (FOXO) [6]. During the process of TAG synthesis, FFAs, one of the major substances, can be obtained from diets or be synthesized *de novo* by two key enzymes, acetyl-CoA carboxylase (ACC) and fatty acid synthase (FAS). The transcription of these two enzymes is controlled by the transcription factor sterol-regulatory element binding protein (SREBP), which can be activated by insulin [1].

The first insect FAS was purified from *D. melanogaster* fat body [7], which was responsible for the biosynthesis of TAG [8]. Later whole-genome shotgun sequence identified three FAS genes in the *Drosophila* genome, and were designated as *FASN1*, *FASN2* and *FASN3,* respectively [9]. Subsequent research demonstrated that FASN1 was involved in glycogen metabolism and TAG biosynthesis, FASN2 was required for the synthesis of branched/methylated long-chain fatty acids that are the precursors of methylated cuticular hydrocarbons, and FASN3 was predicted to be involved in fatty acid biosynthetic process [9–11]. In cotton bollworm *Helicoverpa armigera*, the functions of FAS have not been reported yet, while 14 putative *FAS* genes were predicted from genome sequences, and they were all designated as fatty acid synthase-like (FASL) genes.

Previous studies showed that two insect hormones, juvenile hormone (JH) and 20-hydroxyecdysone (20E), also participate in the regulations of TAG metabolism [12]. JHs are a group of acyclic sesquiterpenoids produced by corpora allata, and they act by directly binding to the basic helix-loop-helix Per-Arnt-Sim (bHLH-PAS) transcription factor Methoprene-tolerant (Met), which can recruit the other bHLH-PAS transcription factor *β*Ftz-F1-interacting steroid receptor coactivator (FISC) and activate the expression of downstream genes [13]. In red flour beetle *Tribolium castaneum*, knockdown of juvenile hormone acid methyltransferase (JHAMT) decreased the level of JH and decelerated lipid catabolism [14]. Knockdown of Met in the cabbage beetle *Colaphellus bowringi* can upregulate the expression of FAS2 and increase the lipid accumulation to cope with stress tolerance and diapause [15]. 20E belongs to steroid hormone and transduces the signals through a heterodimeric nuclear hormone receptor complex comprising Ecdysone receptor (EcR) and Ultraspiracle (USP) [16]. EcR promotes the activation of SREBP, resulting in lipid accumulation in oocytes, and also increases feeding levels in females of *D. melanogaster* [17]. 20E also induces the expression of transcription factor Hepatocyte Nuclear Factor 4 (HNF4), which accelerates the process of β-oxidation, and produces the substances and energy for oocyte development in mosquito *Aedes aegypti* [18].

In addition to insect hormones, microRNAs (miRNAs) are also important regulators of lipid metabolism. The miRNA group is a subcategory of approximately 22 nt non-coding RNAs. They were first identified in *Caenorhabditis elegans* [19], and can bind to the 3′UTR, 5′UTR or coding sequence of target mRNAs to regulate their expression [20–22]. In mammals, numerous miRNAs are proved to modulate lipid metabolism. The miR-33 targets the ATP-binding cassette transporter A1 to depress its translation and decrease the synthesis of high-density lipoprotein in humans and mice [23,24]. FFA-induced miR-122 could be secreted from the liver to blood and reduce TAG levels in mouse liver and muscle tissues by increasing β-oxidation [25]. Human miR-182 inhibits the expression of F-box and tryptophan–aspartic acid repeat domain-containing 7 and CCAAT/enhancer-binding protein *α* leading to reduction of lipid droplets, while glucocorticoids can suppress the transcription of miR-182 [26,27], which implies cross-talking between hormones and lipid-related miRNA. In insects, existing research about miRNA-regulated lipid metabolism has mainly focused on the relationships between miRNA and the insulin pathway. Conserved miR-8 targets to *u-shaped* (a negative regulator of insulin signalling) and increases the body size in *Drosophila* [28,29], while a later report demonstrates this miRNA also promotes lipid accumulation in *A. aegypti* [30]. *Drosophila melanogaster* miR-14 down-regulates the expression of *sugarbabe*, which participates in the synthesis of TAG via insulin-like peptides (*ilp3* and *ilp5*) [31]. Knockout of miR-277 in *A. aegypti* activates the insulin/FOXO signalling by increasing the expression of *ilp7* and *ilp8*, which reduces the level of TAG and inhibits the development of ovaries [32]. However, whether miRNAs can directly modulate the activity of key enzymes in TAG metabolism is still unclear.

In insects, especially holometabolous species, lipid requirements vary greatly among different developmental stages, thus the regulations of lipid metabolism and development are probably relevant. The cotton bollworm, *H. armigera*, is a lepidopterous pest that causes serious economic losses all over the world. Our previous studies have demonstrated that early instar larvae of *H*. *armigera* are more sensitive to entomopathogenic fungi (*Beauveria bassiana*) and an endoparasitoid wasp (*Microplitis mediator*) than later instars [33,34]. Thus, delaying the development of *H*. *armigera* larvae by disrupting lipid synthesis might aid in its population management. In the present study, miR-2055 was found to bind to 3′UTR of fatty acid synthase (*FAS*) and inhibited the translation, which resulted in the reduction of TAG storage and delayed larval development. Further *in vitro* assay demonstrated that the accumulation of miR-2055 could be induced by JH. These data provide an insight into the regulatory mechanisms of both lipid metabolism and larval development in insects, which can help the development of novel pest-control strategies in the future.

## 2. Material and methods

### 2.1. Insects, plants and cell lines

*Helicoverpa armigera* larvae were reared on artificial diet (AD) at 28 ± 1°C under a 14 : 10 h (L : D) photoperiod and 70% relative humidity. The plant *A. thaliana* (AT) Columbia (Col-0) was cultivated at 23 ± 1°C with a 12 : 12 h (L : D) photoperiod and 60% relative humidity. Newly moulted third instar larvae were used in the experiments, and they were reared on AD or fresh leaves of AT until pupation. *Drosophila* S2 cells were cultured in Schneider's Insect Medium (Sigma-Aldrich, USA) containing 10% fetal bovine serum (Thermo Fisher Scientific, USA) at 26 ± 1°C, while *H. armigera* fat body cell (IOZCAS-Ha-I) was maintained in TNM-FH medium (Sigma-Aldrich, USA) with 10% fetal bovine serum under the same conditions.

### 2.2. Sample preparations

Bodyweights of newly moulted third instar larvae were recorded, then they were fed on AD or AT. Weights were recorded again at 1, 2, 4 and 7 days post diet (dpd). Each group contained a total of 48 larvae (fed on AD or AT), and all experiments were performed three independent biological replicates. Fat body samples from ten individuals were dissected from each group for late Nile red staining and total RNA isolation.

### 2.3. Nile red staining

Each sample was washed in phosphate buffered saline (PBS, pH 7.4) three times and was then soaked in Nile red (Sigma-Aldrich, USA) solution (1 µg ml^−1^ in PBS contained 20% glycerol) for 2 h at room temperature. After washing with PBS three times, samples were placed on slides and examined using a confocal laser scanning microscope (Zeiss LSM710, Germany) with an excitation wavelength of 543 nm and an emission wavelength of 626 nm.

### 2.4. RNA isolation, high-throughput sequencing and bioinformatics analysis

Total RNA of fat body was extracted by using TRIzol reagent (Life Technologies, USA) according to the manufacturer's instructions. The mRNA or small RNA enrichment, library construction and high-throughput sequencing were performed by Novogene (Beijing, China) with three biological replications.

Clean data of small RNA (sRNA) with lengths of 18–35 nt were mapped to the genomic sequence of *H. armigera* by using Bowtie [35]. All mapped sRNA was then searched against known miRNAs in miRBASE (http://www.mirbase.org/), and the novel miRNAs were predicted by miREvo and miRDeep2 [36,37]. The miRNA expression level was normalized by transcript per million (TPM) [38], and differential expression analysis was performed by DESeq2 [39]. Target genes of differentially expressed miRNAs were predicted by miRanda and RNAhybrid [40,41]. The abundances of all known and novel transcripts were calculated using featureCounts [42], and differential expression analysis was performed by DESeq2. Differentially expressed transcripts or miRNAs were output when the expression levels in two groups showed a fold change more than 2 and a *p*-value < 0.01.

### 2.5. RT-qPCR analysis of miRNA and mRNA

The reactions for first-strand cDNA synthesis of miRNA and mRNA were conducted using a Mir-X miRNA First-Strand Synthesis Kit (Clontech, USA) and PrimeScript RT reagent Kit with gDNA Eraser (TaKaRa, Japan) according to the manufacturers' instructions, respectively. One microgram of total RNA was used in each reaction, and the synthesized cDNA samples were stored at −20°C for later use. RT-qPCR was carried out in a 10 µl reaction mix containing 5 µl of SYBR Premix Ex Taq (TaKaRa, Japan), 1 µl of cDNA template, 0.5 µl of each of the primers (10 mM; electronic supplementary material, table S1) and 3 µl ddH_2_O. The reactions were performed on a PikoReal 96 Real-Time PCR System (Fisher Scientific, USA). PCR amplification was performed in three independent wells. Relative expression levels were calculated using the 2^−ΔΔCt^ method. The *H. armigera* small nuclear RNA U6 (*HaU6*) and ribosomal protein S3 gene (*HaRPS3*) were set as internal controls for miRNA and mRNA, respectively.

### 2.6. Dual-luciferase reporter assays

A 400 bp pre-miR-2055 centred on *H. armigera* genome sequence was cloned into the pAc5.1/V5-HisB overexpression vector (Invitrogen, USA). The sequence around the binding site of *FAS* 3′ UTR (160 bp upstream and downstream flanks) was inserted into psiCHECK-2 luciferase reporter vector (Promega, USA). The primers are listed in electronic supplementary material, table S1. The sequence in *FAS* that bound to the seed sequence of miR-2055 was mutated by using site mutation based on the principle of adenine/thymine to guanine/cytosine and vice versa. A total of 400 ng of recombinant psiCHECK-2 plasmid (WT or MT) was cotransfected with 400 ng of recombinant pAc5.1/V5-HisB vector overexpressing miR-2055 into *Drosophila* S2 cells by Attractene (Qiagen, Germany), and the luciferase activities were measured at 48 h post transfection using the Dual-Luciferase Reporter Assay System (Promega, USA) with a Glomax Multi^+^ Detection System (Promega, USA).

### 2.7. Injection of agomir

Agomir for miR-2055 was synthesized by Ribo Biotechnology (Guangzhou, China) based on the mature sequence (UAUUCGAGACCUCUGCUGAUCCU), and *C. elegans* miR-67-3p was used as the negative control. About 0.05 nmol agomir was injected into the haemocoel of newly moulted third instar larvae using the Nanoliter 2000 injector (World Precision Instruments, USA). Samples were collected at 4 days post injection.

### 2.8. DsRNA mediated gene knockdown

Double-stranded RNAs (dsRNAs) against *Met, Kr-h1*, *FAS* and *EGFP* were synthesized as described before [33]. About 2 µg dsRNA was injected into the larval haemocoel using a Nanoliter 2000 injector. The fat body was dissected and collected at 4 days post injection. For gene knockdown in the cell line, 2 × 10^6^ IOZCAS-Ha-I cells were plated in each well of a 6-well plate, and 6 µg dsRNA was transfected to each well using a calcium phosphate cell transfection kit (Beyotime, China) according to the manufacturer's instruction.

### 2.9. Determination of TAG and FFA measurements

The fat body was dissected in PBS, frozen with liquid nitrogen and then pulverized. A total of 100 µl PBS containing 0.5% Tween-20 was added to the tube contained 50 mg fat body sample and then homogenized. After that all the samples were incubated at 70°C for 5 min. The TAG level was measured using triglyceride reagent (Sigma-Aldrich, USA) and free glycerol reagent (Sigma-Aldrich, USA) as before [43]. For the determination of FFAs, pulverized samples were incubated in 1 ml extraction solution (2% H_2_SO_4_ in methanol) for 1 h at 80°C. Samples were shaken and then centrifuged at 5000*g* for 10 min, when 0.3 ml hexane and 1.5 ml H_2_O were added. Supernatants were transferred to new tubes and analysed on an Agilent Technologies 6890N GC-5973N mass selective detector to measure the levels of FFAs [18].

### 2.10. Antibody preparation and western blot

The partial sequence of FAS (PKTVITDREPRDD) was synthesized and used to immunize rabbits at GenScript Biotech Corporation (Nanjing, China) to produce the polyclonal antibody against FAS. Protein samples were resolved on 4–15% gradient SDS-polyacrylamide gels and electrotransferred onto PVDF membranes (Invitrogen, USA). Membranes were first incubated with the primary antibody, then with the secondary antibody conjugated with HRP. SuperSignal West Pico Chemiluminescent Substrate (Thermo Fisher Scientific, USA) was used to visualize the immune complexes on the membrane.

### 2.11. Determination of JH and 20E titres

Haemolymph from the larvae of different groups was collected and centrifuged at 500*g* at 4°C for 5 min. The supernatant was transferred to a new glass tube containing 300 µl hexane, followed by vortexing and centrifuging at 5000*g* at 4°C for 10 min. The upper organic layer was transferred to a new glass tube and dried under a nitrogen stream. JH was determined by means of liquid chromatography-triple tandem mass spectrometry (LC-MS/MS/MS) on a Nexera UHPLC LC-30A (Shimadzu, Japan) and SCIEX Triple Quad 4500 (Applied Biosystems, USA) according to the method published before [44]. Titres of 20E in the larval haemolymph were measured using an insect ecdysone ELISA kit purchased from Nuoyajie Corporation (Beijing, China) according to the manufacturer's instructions [45].

### 2.12. *In vitro* fat body culture

Pre-chilled fifth instar larvae were sterilized with 70% ethanol. The fat body was then dissected in sterile PBS and transferred to a 6-well cell culture plate containing 2 ml Grace insect medium (Life Technologies, USA) and different concentrations (2, 5 and 10 µg ml^−1^) of JH (Sigma-Aldrich, USA) or solvent (acetone). Samples were incubated at 27°C for 8 h followed by total RNA isolation.

### 2.13. Statistical analyses

The bubble charts and heatmaps were generated by R v. 3.5 (www.r-project.org/), and other plots were built and analysed using GraphPad Prism v. 6.0 (GraphPad Software, USA). The statistical significance was determined by Student's *t*-test for unpaired comparisons between two different groups, and *p* < 0.05 was regarded as statistically significant.

## 3. Results

### 3.1. Diets affected lipid accumulation and development in larvae

**Figure 1.**
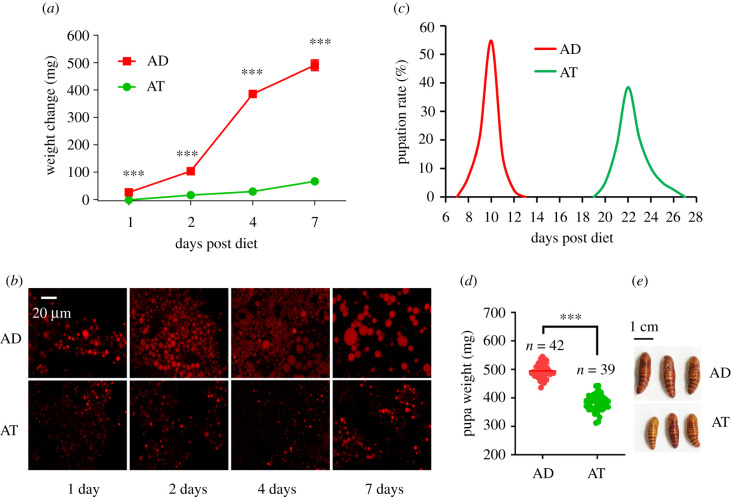
Changes of lipid metabolism and development in *H. armigera* fed on different diets. (*a*) Weight change of *H. armigera* fed on different diets. The body weight of *H. armigera* was measured before treatments and at the first, second, fourth and seventh day after being fed on different diets. The weight change represented the difference between the weight before and after treatments (mean ± s.e.m). (*b*) The fat body of *H. armigera* was stained with Nile red after fed on different diets at the first, second, fouth and seventh day, and examined by the laser scanning microscope. Scale bar, 20 µm. (*c*–*e*) Pupation time (*c*), pupa weight (*d*) and pupa size (*e*) of *H. armigera* fed on different diets. AD, *H. armigera* fed on artificial diet; AT, *H. armigera* fed on *A. thaliana*. ***, *p* < 0.001, *p* < 0.05 indicated the significant difference (Student's *t*-test), and data of pupa weight were shown as mean ± s.e.m.

Diets are important in insect development, but specific mechanisms in the regulatory processes are unclear. To investigate the effects of different diets on larval development, newly moulted third instar *H. armigera* larvae were reared on AD or AT. Results showed that changes in body weight and size of AD and AT were significant at each time point (figure 1*a*; electronic supplementary material, figure S1). At 2 days post diet (dpd), the weight of larvae fed on AD increased more than 100 mg, while the weight of larvae fed on AT only increased about 16 mg ( approx. 6 fold). At 4 dpd, the weight of larvae fed on AD increased more than 380 mg, while the weight of larvae fed on AT only increased about 29 mg (approx. 13 fold). At 7 dpd, the weight of larvae fed on AD increased more than 490 mg, while the weight of larvae fed on AT only increased about 66 mg (approx. 7 fold) (figure 1*a*). Lipid accumulation appeared to play an important role in body weight change, so we dissected the larval fat body at different time points and performed Nile red staining. Images taken by confocal laser scanning microscope showed that lipid droplets in the fat body from larvae fed on AD were much larger than those from larvae fed on AT (figure 1*b*), which was consistent with the changes in body weight.

Notably, at 7 dpd, most larvae fed on AD had reached the wondering stage, while larvae fed on AT retained in the fourth instar (electronic supplementary material, figure S1), which implied the development was delayed in larvae fed on AT. Subsequent pupation rate analysis showed that larvae fed on AT began to pupate at 20 dpd, and completed pupation at 26 dpd, while larvae fed on AD started metamorphosis at 8 dpd, and accomplished this physiological process at 12 dpd (figure 1*c*). After pupation, we weighed all pupae and found that larvae fed on AD formed pupae that weighed about 500 mg, while pupae from AT fed larvae weighed about 380 mg (figure 1*d*). The AT-fed larvae formed smaller pupae than those fed on AD (figure 1*e*). These results indicated larvae fed on AT decelerated lipid synthesis and retarded developmental process significantly compared with larvae fed on AD.

### 3.2. Diets altered transcriptional profiles and hormone levels in larvae

**Figure 2.**
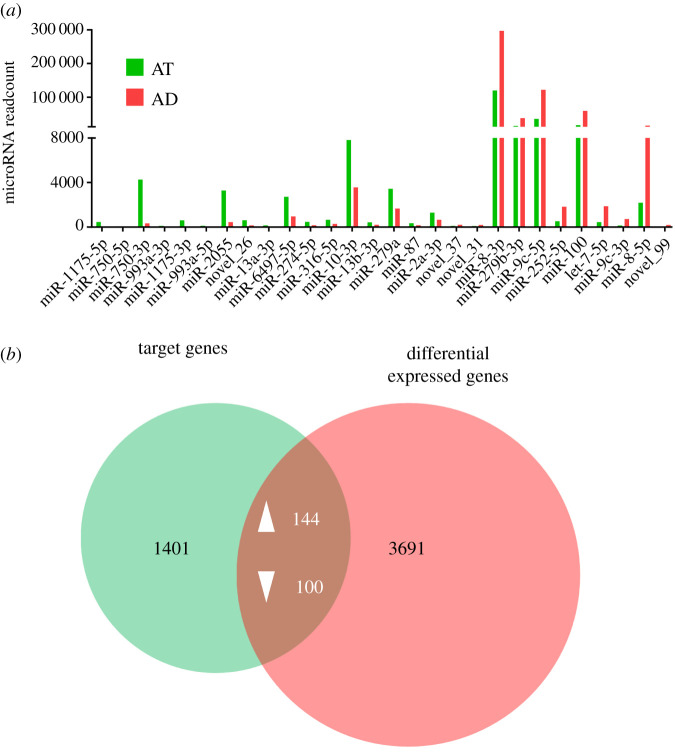
Alteration of microRNA expression profile in *H. armigera* fed on different diets. (*a*) Expression analysis of differentially expressed miRNAs in *H. armigera* fed on different diets. The *x*-axis indicates the 28 differential expressed miRNAs, and the *y*-axis indicates the miRNA expression level. The relative miRNA expression level was represented by the average readcount of three duplicates. (*b*) Venn diagram representation of candidate target genes of DEMs (green circle) and DEGs from transcriptomic data (red circle). Numbers in black type indicate numbers of genes, while numbers in white type indicate numbers of upregulated and downregulated genes.

Since body weight and lipid content showed the most significant differences between larvae fed on AD and AT at 4 dpd, we then performed high-throughput small RNA (sRNA) sequencing to identify certain miRNAs that might play modulating roles in this process. The miRNA expression level was normalized by TPM, and miRNAs with TPM values of more than 50 in at least one group were selected for expression analysis. Results of differential expressed miRNAs (DEMs) showed that 17 miRNAs were upregulated in larvae fed on AT, while 11 miRNAs were downregulated significantly compared with larvae fed on AD (figure 2*a*). To identify key miRNAs and their corresponding regulatory genes that played critical roles in lipid metabolism of *H. armigera*, we performed target gene predictions on all 28 differentially expressed miRNAs. Genes output from both miRanda and RNAhybrid were designated as candidate targets, and 1645 candidates were predicted from the genomic sequence of *H. armigera* totally (figure 2*b*). To search for the key functional genes, transcriptomic sequencing of mRNA in the same samples was conducted accordingly. Expression analysis of differentially expressed genes (DEGs) identified 2191 upregulated genes and 1744 downregulated genes (electronic supplementary material, figure S2). We then generated a Venn diagram with the candidate target genes and the DEGs. Among the candidate genes, 144 genes were upregulated, while 100 genes were downregulated in larvae fed on AT (figure 2*b*).

**Figure 3.**
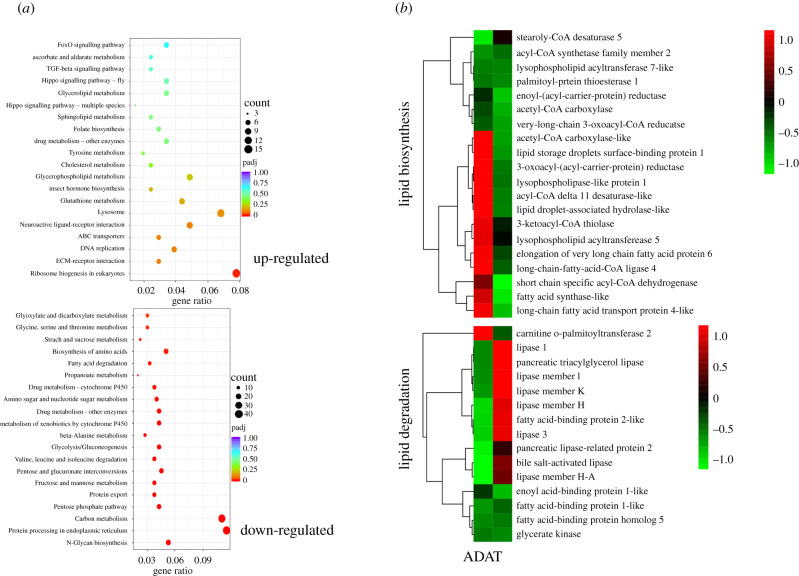
Transcriptomic analysis of DEGs in *H. armigera* fed on different diets. (*a*) KEGG functional enrichment analysis of upregulated genes and downregulated genes. The size of the dots represents the gene number, and the colour represents the *q*-value of the enrichment. Gene ratio is the ratio between the enriched gene number and the total gene number of the corresponding pathway. (*b*) Hierarchical clustering analysis of differentially expressed lipid metabolism-related genes.

To further understand the function of these DEGs, KEGG functional enrichment was conducted and the results showed that genes on the pathways of metabolism and development were enriched significantly (figure 3*a*). We then screened all of the DEGs involved in lipid metabolism and found that 20 genes were related to lipid biosynthesis, while 15 genes were associated with lipid degradation. In order to visualize the expression pattern of these lipid-related genes intuitively, a heat map based on gene abundance was generated, and the results suggested that lipid biosynthesis genes were mostly downregulated, while the majority of the lipid degradation genes were upregulated (figure 3*b*). This was consistent with the lower lipid content in larvae fed on AT.

**Figure 4.**
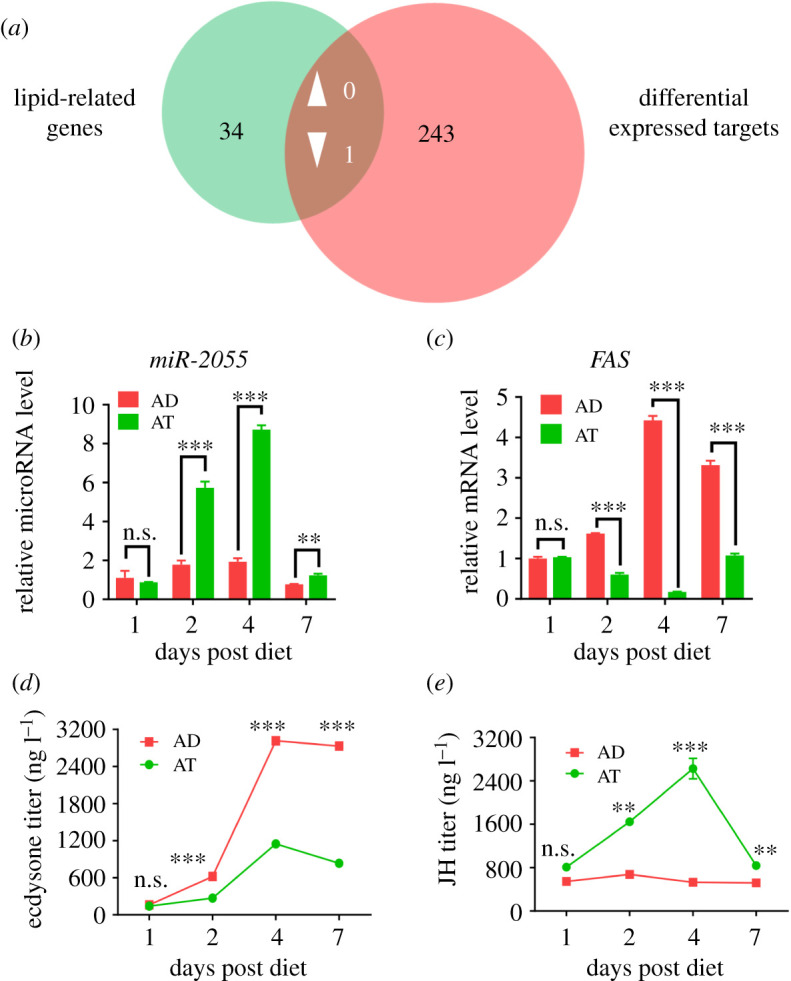
Diets changed expression of lipid-related genes and titres of insect hormones in *H. armigera*. (*a*) Venn diagram representation of lipid-related genes (green circle) and differentially expressed target genes of DEMs (red circle). (*b*,*c*) Expression analysis of miR-2055 (*b*) and *FAS* (*c*) in *H. armigera* fed on different diets. *HaU6* was set as the internal reference for miRNA, and *HaRPS3* was set as the internal reference for the target gene. (*d*,*e*) Quantification of ecdysone (*d*) and JH (*e*) in *H. armigera* fed on different diets. ns, *p* > 0.05; **, *p* < 0.01; ***, *p* < 0.001. *p* < 0.05 indicated the significant difference (Student's *t*-test). Each experiment was performed in three replicates, and data are shown as mean ± s.e.m.

**Figure 5.**
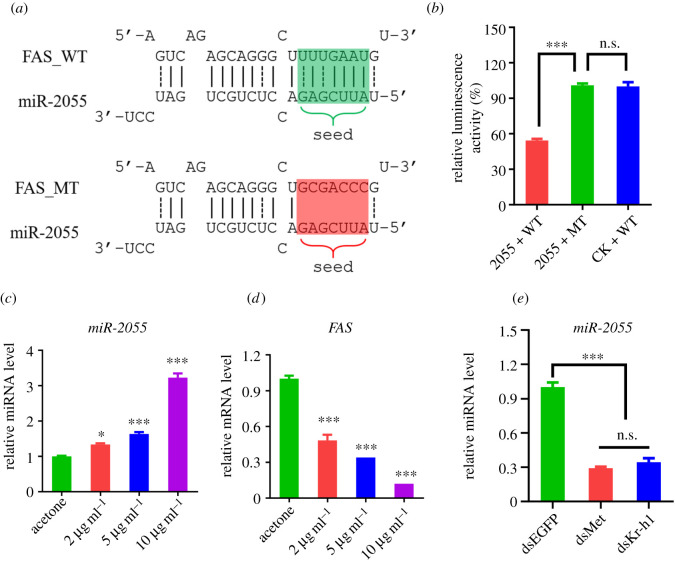
Analysis of expression pattern of miR-2055. (*a*) Sequence alignment of miR-2055 and the predicted target site in the 3′UTR of *FAS*. miR-2055, the mature sequence of miR-2055; FAS_WT, the predicted target site in the 3′UTR of *FAS*; FAS_MT, mutated target site; seed, the seed sequence in the target site. (*b*) Relative luminescence activity in S2 cells transfected with different vectors. 2055 + WT, cells co-transfected with recombinant pAc5.1/V5-His B overexpressing miR-2055 and recombinant psiCHECK inserted with wild-type sequence of *FAS* 3′UTR; 2055 + MT, cells co-transfected with recombinant pAc5.1/V5-His B overexpressing miR-2055 and recombinant psiCHECK inserted with site mutated sequence of *FAS* 3′UTR; CK + WT, cells co-transfected with pAc5.1/V5-His B empty vector and recombinant psiCHECK inserted with wild-type sequence of *FAS* 3′UTR. Each experiment was performed in four replicates. (*c*,*d*) Expression analysis of miR-2055 (*c*) and *FAS* (*d*) in different concentrations of JH. (*e*) Expression analysis of miR-2055 in cell line transfected with different dsRNAs. Each experiment was performed in three replicates. Data were shown as mean ± s.e.m. n.s., *p* > 0.05; *, *p* < 0.05; ***, *p* < 0.001. *p* < 0.05 indicates significant difference.

To identify the key miRNAs and the corresponding target genes that might control the lower TAG level in larvae fed on AT, we conducted intersection between the 35 lipid-related genes and the 244 differential expressed target genes, and found that the only gene that overlapped in the above two groups was LOC110369828, a putative *FASL* gene (figure 4*a*). We then downloaded the protein sequence of FASL in *H. armigera*, as well as other known sequences for FAS in four different Lepidoptera insects from NCBI, and the sequence alignment results showed that HaFASL showed high similarity to known FAS sequences (electronic supplementary material, figure S3), so we designated it as FAS in *H. armigera*. In the 28 differential expressed miRNAs between AD and AT, miR-2055 was predicted to bind to the 3′UTR of *FAS* (figure 5*a*). We checked the expression level of miR-2055 and *FAS* at each time point, and found feeding on AT significantly elevated the expression levels of miR-2055 at 2, 4 and 7 dpd, and the expression level was raised to 4.5 fold at 4 dpd compared with larvae fed on AD (figure 4*b*). For the expression dynamics of *FAS*, the pattern was almost opposite to those of miR-2055 (figure 4*c*), which might imply their negative correlation of expression.

Because different diets changed larval development, we speculated that the two major insect hormones, 20E and/or JH, might have important roles in this process. We determined the titres of 20E and JH in larval haemolymph at 1, 2, 4 and 7 dpd. The results showed that titres of 20E increased from 1 to 4 dpd, and then decreased at 7 dpd in larvae fed on either AD or AT, and the titres in AD were always higher than those in AT (figure 4*d*). JH titres in larvae fed on AD did not change remarkably at different time points; however, in larvae fed on AT, JH titre reached a peak at 4 dpd, and the titres in AT were always higher than those in AD (figure 4*e*). A comparison of the expression pattern of miR-2055 in larvae fed on AD or AT at different time points showed that the expression level of miR-2055 changed along with JH titres, but was not obviously correlated with 20E titres. We also analysed the abundances of JH-related genes based on transcriptomic data, and found that a JH-synthetized gene (aldo-keto reductase) was upregulated significantly in larvae fed on AT, while JH-degraded genes (juvenile hormone esterase, juvenile hormone esterase-like, juvenile hormone epoxide hydrolase and juvenile hormone epoxide hydrolase-like) were significantly downregulated in larvae fed on AT (electronic supplementary material, figure S4), which was consistent with the higher JH titres in AT fed larvae.

### 3.3. miR-2055 bound to 3′UTR of FAS and were regulated by JH signalling pathway

To confirm the association between miR-2055 and its candidate target *FAS*, we conducted a dual luciferase reporter assay. A recombinant psiCHECK-2 vector inserted with wild-type or mutant 3′UTR sequence of *FAS* was constructed (figure 5*a*). Transient expression of miR-2055 in S2 cells with the presence of wild-type 3′UTR sequence of *FAS* decreased luciferase activity to about 50%, but the activity did not change significantly when the seed sequence of *FAS* was mutated (figure 5*b*). These results demonstrated that miR-2055 could repress the expression of FAS by binding to the 3′UTR sequence.

To further verify the effect of JH on the expression of miR-2055, *in vitro* fat body culture was performed. The expression level of miR-2055 increased significantly with addition of JH in the medium (figure 5*c*). Correspondingly, the relative mRNA abundance of *FAS* dropped to only 10% compared with the cultured fat body without JH accordingly (figure 5*d*). We also performed dsRNA mediated knockdown in the fat body cell line of *H. armigera* and found that the expression of miR-2055 dropped to about 30% when the expression of Met or Kr-h1 was interfered (figure 5*e*). These results indicated that JH signalling pathway is critical in the diet-induced metabolism and developmental differences by increasing the accumulation of miR-2055 via its receptor Met, and Kr-h1 is also involved.

### 3.4. miR-2055 decreased FFAs level and resulted in TAG insufficiency

**Figure 6.**
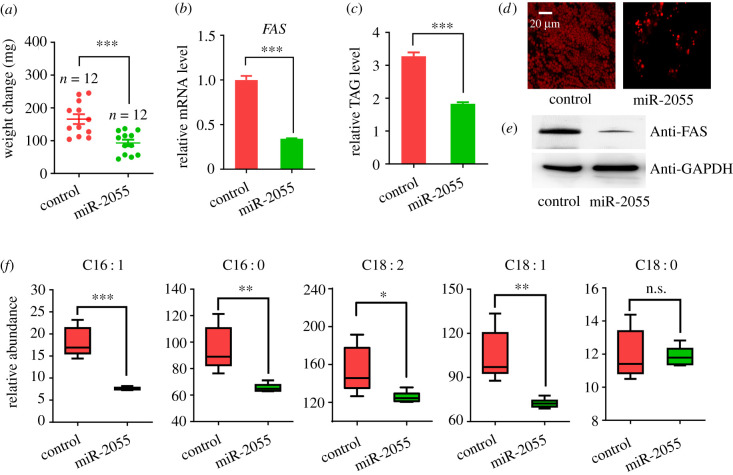
Effects of miR-2055 agomir on lipid biosynthesis in *H. armigera.* Body weight change (*a*), expression analysis of *FAS* (*b*), relative TAG level (*c*), Nile red stain of lipid droplets (*d*), immunoblot analysis (*e*) and abundance of FFAs (*f*) in *H. armigera* after agomir injection. Agomir for negative control or miR-2055 was injected into the hemocoel of newly moulted third instar larvae, and fat body was dissected at 4 days post injection. ns, *p* > 0.05; *, *p* < 0.05; **, *p* < 0.01; ***, *p* < 0.001; scale bar, 20 µm. Polyclonal antibody against FAS (1 : 8000) was used to detect the target protein (upper). GAPDH was set as the internal reference (1 : 5000, bottom). C16 : 1, 9-hexadecenoic acid; C16 : 0, hexadecanoic acid; C18 : 2, 9,12-octadecadienoic acid; C18 : 1, 11-octadecenoic acid; C18 : 0, octadecanoic acid. Experiments of RT-qPCR and TAG assay were performed in three replicates (mean ± SEM), while the experiment of FFA determination was performed in six replicates. *p* < 0.05 indicated the significant difference.

To substantiate the roles of miR-2055, miRNA agomir was synthesized *in vitro* based on its mature sequence and injected into the larval hemocoel. Four days post injection, the body weight of larvae injected with miR-2055 agomir increased about 93 mg, while larvae injected with control agomir increased about 166 mg (figure 6*a*). RT-qPCR demonstrated the mRNA level of *FAS* fell to about 40% after miR-2055 agomir injection (figure 6*b*). Western blot results also showed a remarkable reduction of FAS in larvae injected with miR-2055 agomir (figure 6*e*). In addition, the relative TAG level and the number of lipid droplets both decreased dramatically (figure 6*c* and *d*). FAS is the key enzyme in the synthesis of fatty acids, so we determined the content of FFAs in the larval fat body. As expected, the relative abundances of 9-hexadecenoic acid, hexadecanoic acid, 9,12-octadecadienoic acid, and 11-octadecenoic acid were reduced significantly (figure 6*f*). Thus, miR-2055 downregulated the level of FAS at both transcription and translation, which suppressed the synthesis of fatty acids and led to TAG insufficiency in *H. armigera*.

To further confirm the function of FAS in lipid anabolism, dsRNA mediated knockdown was performed. The expression level of FAS was extremely reduced on both mRNA and protein levels (electronic supplementary material, figure S5A and S5B). The average weight gain in *FAS* interfered larvae was only 70% compared with larvae injected with *EGFP* dsRNA (electronic supplementary material, figure S5C). The TAG level was reduced about 30% at 4 days post *FAS* knockdown, and the number of lipid droplets was also decreased notably (electronic supplementary material, figure S5D and S5E). After determination of FFAs in the larval fat body, we found that dsRNA mediated knockdown of *FAS* significantly downregulated the abundances of 9-hexadecenoic acid, hexadecanoic acid, 9,12-octadecadienoic acid, 11-octadecenoic acid and octadecanoic acid (electronic supplementary material, figure S6). Altogether, these results suggest that FAS is essential in the synthesis of fatty acids and TAG.

## 4. Discussion

Insect hormones and miRNAs are major factors involved in modulating lipid metabolism and development, but knowledge of the cross-talking between them is still limited. Here, we found that JH could increase the accumulation of miR-2055 via its receptor Met, and this microRNA inhibited *FAS* expression to block TAG synthesis in the fat body and delay larval development. *H. armigera* is a holometabolous insect that has four distinct developmental stages in its life cycle. The larval stage needs to acquire and store sufficient lipid reserves for reproduction during adult. Thus, regulation of lipid metabolism is essential for not only growth and development but also their adaptive evolution.

Stored lipid in insects is mainly made up of TAG, which is synthesized from FFAs and glycerol, and FFAs are derived from food digestion and *de novo* synthesis using dietary compounds. In other words, food determines what the herbivores and predators are. In order to maximize the fitness to certain hosts, many plant-eating insects even change their genetic background. *Drosophila sechellia* has evolved to tolerate the fruit of *Morinda citrifolia*, which is toxic to other members of the *melanogaster* species [46,47]. Later genetic analysis revealed that a 45 bp deletion (relative to *D. melanogaster*) in the open reading frame of *catsup*, one of the negative regulators in the production of L-DOPA, enables *D. sechellia* to produce larger eggs and drives them to adapt the chemical compounds of *M. citrifolia* [48,49]. To neutralize phenolic glucosides in its host plant, the sweet potato whitefly *Bemisia tabaci* acquires *BtPMaT1*, a plant-specific gene encoding a phenolic glucoside malonyltransferase that enables whiteflies to survive on the flavonoid-rich plant [50].

Compared with long-term co-evolution, a temporary alteration of food supply may impede the metabolism and development of insects. Food shift from a natural host (black-eyed bean, *Vigna unguiculata*) to a novel plant (chick peas, *Cicer arietinum*) in bruchid beetle (*Callosobruchus maculatus*) prolonged the development time significantly [51]. Similar research on *C. maculatus* also found that the number of eggs laid by each female beetle decreased when they were moved to the other host [52]. These studies revealed host adaptations of herbivorous insects and provided insights on the aspect of evolution. However, the molecular details remained limited. In this study, we fed *H. armigera*, a worldwide lepidopteran pest, with distinct foods, and attributed the changes in lipid metabolism and development to miRNA and JH.

MiRNAs are nucleotide sequences around 22 nt long that possess a regulatory role in diverse biological processes [53]. The first description of miRNA in metabolic processes in animals was miR-14 in *D. melanogaster*, which could suppress Reaper-dependent cell death and decrease the level of TAG [54]. Later research present the correlation between miRNA and lipid metabolism in insects. Depletion of miR-8 in mosquitoes by antagomir injection could upregulate its target gene secreted wingless-interacting molecule and decrease the level of lipid in female ovaries to block vitellogenesis post blood meal [30]. *A. aegypti* miR-277 targets and downregulates *ilp7* and *ilp8* to block the insulin/FOXO signalling and promote TAG synthesis [32]. In this study, we demonstrated that *H. armigera* miR-2055 can bind to 3′UTR of *FAS* to inhibit its expression and lead to the reduction of FFAs and TAG levels in the larval fat body (figures 5 and 6). Furthermore, we also present that JH acts as the activator of miR-2055 (figure 5*c* and *e*), which bridges the regulatory roles of JH and miRNA in lipid metabolism and larval development.

Since chemical pesticides has caused serious damage to agricultural products as well as to the environment, and susceptibility of *H. armigera* to the *Bacillus thuringiensis* crystal protein has decreased in spore and plant-based products [55]. The discovery and development of novel biological pesticides are needed. Small RNAs are important regulatory elements in post-transcriptional modifications, and have shown some potential in pest control. Feeding larvae with plants that expressing dsRNA specific to *HaCYP6AE14* downregulates its expression level in the midgut and leads to lower tolerance to gossypol in *H. armigera* [56]. Transplastomic potato plants producing dsRNAs targeting against the *β-*actin of the Colorado potato beetle are lethal to its larvae [57]. In addition to dsRNAs, miRNAs also present great abilities in the development of novel pesticides. Feeding with *Escherichia coli* expressing the let-7a precursor miRNA silences *EcR* and leads to developmental disorders in *H. armigera* [58]. In rice stem borer, *Chilo suppressalis*, bantam and miR-9b target to genes in the ecdysteroid biosynthesis pathway and reduce the titre of 20E, and injection of these miRNAs retards larval development [59]. In this study, we demonstrated that miR-2055 in *H. armigera* could delay larval development by inhibiting lipid biosynthesis, which suggested that miR-2055 can be used as a new target to achieve environmentally friendly pesticide.

Previous studies have revealed that JH acts as an antagonist in lipid synthesis. In *Culex pipiens*, FOXO increased lipid reserves in the fat body of diapause females to keep them alive, while JH could suppress the expression of FOXO [60]. RNAi-aided knockdown of JHAMT reduced JH biosynthesis and resulted in the rise of lipid in starved *T. castaneum* [14]. Knockdown of *Bmm* in brown planthopper, *Nilaparvata lugens*, resulted in an increase of JH esterase and a decline of JH receptors as well as their downstream transcription factors, which led to obesity and blocked lipid mobilization in the fat body [61]. In the present study, we used different foods to feed cotton bollworm and reveal that AT-diet increases JH titre before 4 dpd (figure 4*e*) leading to the reduction of lipid accumulation, which indicates the antagonism of JH to lipid synthesis, consistent with previous findings. In the meantime, we step further and demonstrate that JH can also control the expression of the key enzyme in lipid metabolism via miRNA as intermediates, which has not been reported previously.

Researchers had isolated JH III from grasshopper's cyperus (*Cyperus iria*), and this plant-derived JH III showed the same 10R configuration as insect-secreted JH III. Grasshopper (*Melanoplus sanguinipes*) nymphs fed on *C. iria* presented typical metatheteletic effects like those produced by JH treatment [62]. The following report demonstrated that later steps of JH III biosynthesis in *C. iria* were similar to those in the insect pathway [63]. These implied that insects could uptake plant-derived JH directly and/or synthesize JH using plant compounds obtained from plant feeding. Our results also showed that plant feeding increased JH titre dramatically and changed the expression pattern of JH-related genes (figure 4*e*; electronic supplementary material, figure S4), however, it remains to be elucidated whether the excess JH is absorbed directly from AT leaves or synthesized in larvae using plant compounds as precursors.

## Data Availability

The data of high-throughput mRNA and small RNA sequencing described in this manuscript are available under the BioProject accession no. PRJNA699340. Illumina sequence reads have been deposited in the NCBI SRA database under the following accession numbers (mRNA of fat body from larvae fed on AD: SRR13625115, SRR13625118, SRR13625119; mRNA of fat body from larvae fed on *A. thaliana*: SRR13625112, SRR13625113, SRR13625114; small RNA of fat body from larvae fed on AD: SRR13625109, SRR13625110, SRR13625111; small RNA of fat body from larvae fed on *A. thaliana*: SRR13625108, SRR13625116, SRR13625117). These data are also deposited into Science Data Bank as the accession no. 31253.11.sciencedb.o00019.00003.
